# Supplementary material for: GEsture: an online hand-drawing tool for gene expression pattern search
Source: PeerJ. 2018 Jun 20;6:e4927. doi: 10.7717/peerj.4927 (PMC6015481; doi:10.7717/peerj.4927)
Supplement: Table S1 — In the column Search Pattern, the numbers in the bracket represent the count of genes searched by GEsture. [file peerj-06-4927-s001.docx]

| **Search Pattern** | **Genes** |
| --- | --- |
| **Similar pattern**  **(155)** | YOR074C,YML027W,YBR088C,YLR103C,YDL164C,  YGR152C,YKL113C,YER070W,YHR110W,YPL256C,  YDL003W,YNL312W,YDL018C,YER095W,YGR151C,  YPL153C,YDL156W,YGR189C,YDL163W,YPR135W,  YNL231C,YDL010W,YOR321W,YOL090W,YPL221W,  YNL300W,YKL067W,YDR400W,YPL208W,YNL309W,  YPR175W,YPR120C,YDL009C,YJL074C,YGR109C,  YJR006W,YDL103C,YMR029C,YMR078C,YGR221C,  YJL091C,YPL163C,YLR183C,YER111C,YMR179W,  YJL181W,YLR050C,YBR070C,YNL072W,YOL147C,  YPR174C,YBR161W,YOR033C,YNL181W,YKL066W,  YJL073W,YER170W,YDR507C,YKL045W,YJL115W,  YDR097C,YHR149C,YAR007C,YKL165C,YDL101C,  YFL062W,YCR065W,YLR465C,YGR286C,YER071C,  YGL163C,YCL061C,YNL339C,YGR238C,YDL093W,  YMR075W,YNL082W,YHR160C,YHR188C,YIR043C,  YPR202W,YOR195W,YNR077C,YPL267W,YMR095C,  YPL057C,YKL101W,YFR027W,YPL255W,YFL060C,  YHL048W,YPL015C,YDR503C,YML060W,YOL008W,  YOL017W,YEL064C,YCL022C,YCL024W,YJL019W,  YDR013W,YHR159W,YNL233W,YJL201W,YDR279W,  YBR041W,YFR041C,YFL061W,YLR326W,YNL165W,  YIL139C,YBR087W,YDR518W,YDR440W,YKL046C,  YLR018C,YLR464W,YPR204W,YLR467W,YFL059W,  YDR040C,YLR457C,YOR176W,YGL175C,YFR042W,  YJL097W,YKR090W,YOR317W,YIL147C,YBL035C,  YGL198W,YPR076W,YMR094W,YGL062W,YLR236C,  YAR003W,YBR149W,YPL241C,YEL077C,YNL130C,  YKR012C,YGR019W,YFR053C,YER118C,YGR296W,  YDR348C,YPL017C,YDR538W,YHR071W,YNL336W,  YDR113C,YLR463C,YHL050C,YDR501W,YLR462W |
| **Contrast**  **Pattern**  **(15)** | YOR229W,YMR217W,YPR156C,YLR254C,YCL005W, YOR059C,YDR061W, YDR087C, YLR146C,YLR180W,  YOL056W, YCRX12W, YLR003C, YDR346C, YKL069W |
| **Shift pattern**  **(44)** | YLR326W,YDR518W,YDR414C,YOR176W,YDR440W,  YOR378W,YPR076W,YOR039W,YHL049C,YJL225C,  YFL068W,YHR218W,YBR071W,YFL066C,YFL067W,  YLR466W,YER189W,YFL064C,YDR528W,YEL076C,  YKL108W,YEL075C,YBL112C,YBR001C,YDR545W  YJR066W,YOR111W,YOR317W,YIR003W,YJR043C  YOR132W,YDR508C,YDR279W,YJR127C, YBR287W,  YIL170W, YPL061W, YIR036C, YHR057C, YHR057C,  YER183C, YIR034C, YDL194W, YDL194W |
