# Supplementary material for: GEsture: an online hand-drawing tool for gene expression pattern search
Source: PeerJ. 2018 Jun 20;6:e4927. doi: 10.7717/peerj.4927 (PMC6015481; doi:10.7717/peerj.4927)
Supplement: Table S2 — Concrete target genes of three search patterns corresponding to drawing a curve like the expression of YNL309W are listed separately in the table. The gene name labeled red color represents that these genes are regulated by both TEC1p and STE12p. [file peerj-06-4927-s002.docx]

| **Pattern** | **TF(s)** | **Genes** |
| --- | --- | --- |
| **Similar pattern** | TEC1p | YKL113C,YER070W,YHR110W,YDL003W,YDL156W,  YDL010W,YOR321W,YDR400W,YMR078C,YJL091C,  YJL181W,YNL072W,YPR174C,YFL062W,YNL339C,  YGR238C,YDL093W,YPL267W,YMR095C,YFR027W,  YDR013W,YNL233W,YJL201W,YBR041W,YNL165W,  YKL046C,YLR467W,YBL035C,YPR076W,YBR149W,  YKR012C,YDR113C,YHL050C，YOR074C,YML027W,YBR088C,YLR103C,YDL164C,  YGR152C,YPL256C,YPL153C,YGR189C,YDL163W,  YPR135W,YOL090W,YNL300W,YNL309W,YPR175W,  YPR120C,YJL074C,YGR109C,YMR029C,YGR221C,  YPL163C,YLR183C,YER111C,YMR179W,YBR161W,  YOR033C,YJL073W,YER170W,YDR507C,YKL045W,  YJL115W,YDR097C,YHR149C,YAR007C,YKL165C,  YDL101C,YCR065W,YLR465C,YGR286C,YER071C,  YGL163C,YCL061C,YMR075W,YNL082W,YHR160C,  YPR202W,YOR195W,YKL101W,YPL255W,YHL048W,  YML060W,YOL017W,YCL024W,YJL019W,YHR159W,  YDR279W,YFR041C,YLR326W,YIL139C,YDR440W,  YLR464W,YFL059W,YDR040C,YLR457C,YOR176W,  YGL175C,YFR042W,YIL147C,YMR094W,YGL062W,  YPL241C,YFR053C,YER118C,YNL336W,YLR463C,  YLR462W |
|  | STE12p | YER095W,YGR151C,YPL208W,YJR006W,YKL066W,  YNR077C,YPL057C,YPL015C,YDR503C,YEL064C,  YKR090W,YOR317W,YGR296W,YHR071W,YDR501W，  YOR074C,YML027W,YBR088C,YLR103C,YDL164C,  YGR152C,YPL256C,YPL153C,YGR189C,YDL163W,  YPR135W,YOL090W,YNL300W,YNL309W,YPR175W,  YPR120C,YJL074C,YGR109C,YMR029C,YGR221C,  YPL163C,YLR183C,YER111C,YMR179W,YBR161W,  YOR033C,YJL073W,YER170W,YDR507C,YKL045W,  YJL115W,YDR097C,YHR149C,YAR007C,YKL165C,  YDL101C,YCR065W,YLR465C,YGR286C,YER071C,  YGL163C,YCL061C,YMR075W,YNL082W,YHR160C,  YPR202W,YOR195W,YKL101W,YPL255W,YHL048W,  YML060W,YOL017W,YCL024W,YJL019W,YHR159W,  YDR279W,YFR041C,YLR326W,YIL139C,YDR440W,  YLR464W,YFL059W,YDR040C,YLR457C,YOR176W,  YGL175C,YFR042W,YIL147C,YMR094W,YGL062W,  YPL241C,YFR053C,YER118C,YNL336W,YLR463C,  YLR462W |
| **Contrast pattern** | TEC1p | YPR156C,YLR254C,YCL005W |
|  | STE12p | YOR229W,YMR217W,YPR156C,YLR254C,YCL005W |
| **Shift pattern** | TEC1p | YPR076W,YER189W,YJR066W, YIR003W,YFL067W,  YLR326W,YOR176W,YDR440W,YOR378W,YHL049C,  YJL225C,YFL068W,YHR218W,YBR071W,YFL064C,  YDR528W,YEL076C,YKL108W,YEL075C,YDR545W,  YOR111W,YJR043C,YDR508C,YDR279W,YJR127C |
|  | STE12p | YBR001C,YOR317W,  YLR326W,YOR176W,YDR440W,YOR378W,YHL049C,  YJL225C,YFL068W,YHR218W,YBR071W,YFL064C,  YDR528W,YEL076C,YKL108W,YEL075C,YDR545W,  YOR111W,YJR043C,YDR508C,YDR279W,YJR127C |
