# Supplementary material for: GEsture: an online hand-drawing tool for gene expression pattern search
Source: PeerJ. 2018 Jun 20;6:e4927. doi: 10.7717/peerj.4927 (PMC6015481; doi:10.7717/peerj.4927)
Supplement: Table S3 — The table lists the functions of circadian rhythm genes sought out by the three search patterns. P-value and corr represent the correlation between the gene we found and the curve. And more gene information can be introduced in the website of TAIR. [file peerj-06-4927-s003.docx]

| **Search**  **pattern** | **Gene name** | **P_value** | **Corr** | **Description** |
| --- | --- | --- | --- | --- |
| **Similar pattern** | AT5G02840 | 0.007420807 | 0.72673176 | Encodes RVE4, a homolog of the circadian rhythm regulator RVE8. rve4 rve6 rve8 triple mutants display an extremely long circadian period, with delayed and reduced expression of evening-phased clock genes. |
|  | AT5G25830 | 0.006033446 | 0.739001489 | Encodes a member of the GATA factor family of zinc finger transcription factors. |
|  | AT4G24500 | 0.011316617 | 0.699644569 | Encodes a proline-rich protein SICKLE (SIC). Required for development and abiotic stress tolerance. Involved in microRNA biogenesis. It is involved in mRNA splicing. It is a single copy gene in Arabidopsis and likely specific to higher plants. Along with RCN1, it functions in regulating auxin transport processes in part by regulating the recycling of PIN1 and PIN2 auxin transporters. It is required for circadian clock temperature responses. |
|  | AT5G67380 | 0.001133205 | 0.818519783 | Casein kinase II (CK2) catalytic subunit (alpha 1). One known substrate of CK2 is Phytochrome Interacting Factor 1 (PIF1). CK2-mediated phosphorylation enhances the light-induced degradation of PIF1 to promote photomorphogenesis. |
|  | AT5G15850 | 0.012449507 | 0.693104362 | Homologous to the flowering-time gene CONSTANS. |
|  | AT3G57040 | 0.000272591 | 0.865787456 | response regulator ARR9, A two-component response regulator-like protein with a receiver domain with a conserved aspartate residue and a possible phosphorylation site and at the N-terminal half. Appears to interact with histidine kinase like genes ATHP3 and ATHP2 |
|  | AT5G05660 | 0.005948853 | 0.739815795 | Encodes a homolog of the mammalian zinc finger transcription factor NF-X1. |
|  | AT2G18915 | 0.000228649 | 0.870644557 | encodes a member of F-box proteins that includes two other proteins in Arabidopsis (ZTL and FKF1). These proteins contain a unique structure containing a PAS domain at their N-terminus, an F-box motif, and 6 kelch repeats at their C-terminus. Overexpression results in arrhythmic phenotypes for a number of circadian clock outputs in both constant light and constant darkness, long hypocotyls under multiple fluences of both red and blue light, and a loss of photoperiodic control of flowering time. Although this the expression of this gene itself is not regulated by circadian clock, it physically interacts with Dof transcription factors that are transcriptionally regulated by circadian rhythm. LKP2 interacts with Di19, CO/COL family proteins. |
|  | AT3G50000 | 0.001325955 | 0.812310735 | Encodes the casein kinase II (CK2) catalytic subunit (alpha). |
|  | AT1G80820 | 0.008790559 | 0.716205727 | Encodes an cinnamoyl CoA reductase isoform. Involved in lignin biosynthesis. |
|  | AT5G56860 | 0.00373952 | 0.765076915 | Encodes a member of the GATA factor family of zinc finger transcription factors. Modulate chlorophyll biosynthesis and glutamate synthase (GLU1/Fd-GOGAT) expression. |
|  | AT2G47700 | 0.009470357 | 0.711432395 | RING/U-box superfamily protein;(source:Araport11) |
|  | AT3G55960 | 0.008300426 | 0.719821263 | Haloacid dehalogenase-like hydrolase (HAD) superfamily protein;(source:Araport11) |
|  | AT3G56480 | 0.016955358 | 0.670777213 | myosin heavy chain-like protein;(source:Araport11) |
| **Contrast**  **pattern** | AT3G06500 | 7.01E-05 | 0.898885441 | Encodes an alkaline/neutral invertase which localizes in mitochondria. It may be modulating hormone balance in relation to the radicle emergence. Mutants display severely reduced shoot growth and reduced oxygen consumption. Mutant root development is not affected as reported for A/N-InvA mutant (inva) plants. The mRNA is cell-to-cell mobile |
|  | AT3G46640 | 0.000540151 | 0.844982776 | Encodes a myb family transcription factor with a single Myb DNA-binding domain (type SHAQKYF) that is unique to plants and is essential for circadian rhythms, specifically for transcriptional regulation within the circadian clock. LUX is required for normal rhythmic expression of multiple clock outputs in both constant light and darkness. It is coregulated with TOC1 and seems to be repressed by CCA1 and LHY by direct binding of these proteins to the evening element in the LUX promoter. The mRNA is cell-to-cell mobile. |
|  | AT2G42540 | 0.005906864 | 0.740223224 | A cold-regulated gene whose product is targeted to the chloroplast. Cor15am protects stromal proteins from aggregation under various stress conditions. Constitutive expression increases freezing tolerance in protoplasts in vitro and chloroplasts in vivo. NMR and x-ray diffraction studies suggest that COR15a alters the intrinsic curvature of the inner membrane of chloroplast envelope. Late Embryogenesis abundant protein (LEA). Protects chloroplast membranes during freezing |
|  | AT4G02630 | 0.00074507 | 0.834040336 | Protein kinase superfamily protein;(source:Araport11) |
|  | AT3G52180 | 0.000111093 | 0.888738822 | Encodes a plant-specific glucan phosphatase that contains a noncatalytic carbohydrate-binding module as well as a dual specificity protein phosphatase domain. SEX4 can dephosphorylate C6- and C3-glucosyl residues on native starch grains and related maltodextrin compounds in vitro. This protein interacts with the plant SnRK AKIN11, binds starch, and is localized in the chloroplast. sex4 mutants have elevated levels of starch. |
|  | AT1G68050 | 0.002134957 | 0.79203872 | Encodes FKF1, a flavin-binding kelch repeat F box protein, is clock-controlled, regulates transition to flowering. Forms a complex with GI on the CO promoter to regulate CO expression. |
|  | AT4G26700 | 0.010693177 | 0.703453511 | fimbrin 1;(source:Araport11) |
|  | AT2G21660 | 0.000113376 | 0.888266784 | Encodes a small glycine-rich RNA binding protein that is part of a negative-feedback loop through which AtGRP7 regulates the circadian oscillations of its own transcript. Gene expression is induced by cold. GRP7 appears to promote stomatal opening and reduce tolerance under salt and dehydration stress conditions |
|  | AT3G46780 | 0.003003238 | 0.776055105 | plastid transcriptionally active 16;(source:Araport11) |
|  | AT5G61380 | 0.011770798 | 0.696966715 | Pseudo response regulator involved in the generation of circadian rhythms. TOC1 appears to shorten the period of circumnutation speed. TOC1 contributes to the plant fitness (carbon fixation |
|  | AT2G18170 | 0.00975461 | 0.709512078 | MAP kinase 7;(source:Araport11) |
| **Shift pattern** | AT4G24470 | 0.000988393 | 0.847464825 | ZIM is a putative transcription factor containing an atypical GATA-type zinc-finger motif. |
|  | AT4G25100 | 0.024084379 | 0.670029412 | Fe-superoxide dismutase |
|  | AT4G30350 | 0.0034517 | 0.794964266 | Encodes a member of an eight-gene family (SMAX1 and SMAX1-like) that has weak similarity to AtHSP101 |
|  | AT3G22170 | 0.023647405 | 0.67155013 | A component of the PHYA signaling network |
|  | AT1G10470 | 0.01974376 | 0.68611115 | response regulator 4;(source:Araport11) |
|  | AT5G02120 | 0.000516977 | 0.868870233 | Encodes a one helix protein homologous to cyanobacterial high-light inducible proteins. The protein is localized to the thylakoid membrane and its transcript is transiently induced by exposure to high light conditions. The mRNA is cell-to-cell mobile |
|  | AT1G27450 | 0.009055322 | 0.741151716 | Adenosine phosphoribosyl transferase(E.C:2.4.2.7), involved in the one-step salvage of adenine to AMP. |
|  | AT5G37260 | 0.004538128 | 0.781060541 | Encodes a MYB family transcription factor Circadian 1 (CIR1). Involved in circadian regulation in Arabidopsis. |
|  | AT1G15950 | 0.001163789 | 0.841507341 | Encodes a cinnamoyl CoA reductase. Involved in lignin biosynthesis. The mRNA is cell-to-cell mobile. |
|  | AT5G59560 | 0.009777586 | 0.736244264 | Encodes a novel protein conserved in higher eukaryotes. Normal function of the protein is required for normal oscillator function during circadian rhythm. Mutant analyses also suggest a role in phytochrome B (phyB)-mediated light signaling. |
|  | AT3G07650 | 0.011502012 | 0.725511331 | This gene belongs to the CO (CONSTANS) gene family. This gene family is divided in three subgroups: groups III, to which COL9 belongs, is characterised by one B-box (supposed to regulate protein-protein interactions) and a second diverged zinc finger. COL9 downregulates expression of CO (CONSTANS) as well as FT and SOC1 which are known regulatory targets of CO. The mRNA is cell-to-cell mobile. |
|  | AT4G09970 | 0.004241596 | 0.784586675 | transmembrane protein;(source:Araport11) |
|  | AT2G25930 | 0.008873811 | 0.742429599 | Encodes a nuclear protein that is expressed rhythmically and interacts with phytochrome B to control plant development and flowering through a signal transduction pathway. Required component of the core circadian clock regardless of light conditions. |
|  | AT4G34680 | 0.015354857 | 0.70518862 | Encodes a member of the GATA factor family of zinc finger transcription factors. |
|  | AT3G61070 | 0.009784817 | 0.73619649 | member of the peroxin11 (PEX11) gene family, integral to peroxisome membrane, controls peroxisome proliferation. |
